# Designing Equitable Antiretroviral Allocation Strategies in Resource-Constrained Countries

**DOI:** 10.1371/journal.pmed.0020050

**Published:** 2005-02-22

**Authors:** David P Wilson, Sally M Blower

**Affiliations:** 1Department of Biomathematics and UCLA AIDS Institute, School of MedicineUniversity of California, Los Angeles, CaliforniaUnited States of America; St. Vincent's HospitalAustralia

## Abstract

**Background:**

Recently, a global commitment has been made to expand access to antiretrovirals (ARVs) in the developing world. However, in many resource-constrained countries the number of individuals infected with HIV in need of treatment will far exceed the supply of ARVs, and only a limited number of health-care facilities (HCFs) will be available for ARV distribution. Deciding how to allocate the limited supply of ARVs among HCFs will be extremely difficult. Resource allocation decisions can be made on the basis of many epidemiological, ethical, or preferential treatment priority criteria.

**Methods and Findings:**

Here we use operations research techniques, and we show how to determine the optimal strategy for allocating ARVs among HCFs in order to satisfy the equitable criterion that each individual infected with HIV has an equal chance of receiving ARVs. We present a novel spatial mathematical model that includes heterogeneity in treatment accessibility. We show how to use our theoretical framework, in conjunction with an equity objective function, to determine an optimal equitable allocation strategy (OEAS) for ARVs in resource-constrained regions. Our equity objective function enables us to apply the egalitarian principle of equity with respect to access to health care. We use data from the detailed ARV rollout plan designed by the government of South Africa to determine an OEAS for the province of KwaZulu–Natal. We determine the OEAS for KwaZulu–Natal, and we then compare this OEAS with two other ARV allocation strategies: (i) allocating ARVs only to Durban (the largest urban city in KwaZulu–Natal province) and (ii) allocating ARVs equally to all available HCFs. In addition, we compare the OEAS to the current allocation plan of the South African government (which is based upon allocating ARVs to 17 HCFs). We show that our OEAS significantly improves equity in treatment accessibility in comparison with these three ARV allocation strategies. We also quantify how the size of the catchment region surrounding each HCF, and the number of HCFs utilized for ARV distribution, alters the OEAS and the probability of achieving equity in treatment accessibility. We calculate that in order to achieve the greatest degree of treatment equity for individuals with HIV in KwaZulu–Natal, the ARVs should be allocated to 54 HCFs and each HCF should serve a catchment region of 40 to 60 km.

**Conclusion:**

Our OEAS would substantially improve equality in treatment accessibility in comparison with other allocation strategies. Furthermore, our OEAS is extremely different from the currently planned strategy. We suggest that our novel methodology be used to design optimal ARV allocation strategies for resource-constrained countries.

## Abstract 2

A mathematical model that allows to determine a strategy for distribution of limited medical resources (in this case antiretrovirals) such that each individual in need will have an equal chance of receiving treatment

## Introduction

The HIV/AIDS epidemic is having a devastating impact in sub-Saharan Africa and other resource-constrained regions. Recently, the World Health Organization and other organizations have committed to expand access to antiretrovirals (ARVs) in the developing world, the United States government has pledged to provide $15 billion for AIDS in Africa and the Carribean, and drug prices have fallen [1]. However, even if these resources are provided for the global treatment of HIV, the number of individuals in need of treatment will far exceed the supply of ARVs [1]. Thus, difficult decisions will have to be made as to how to design HIV treatment strategies with these scarce resources. Resource allocation decisions can be made on the basis of many different epidemiological, ethical, or preferential treatment priority criteria. Many diverse groups have been suggested for treatment priority in resource-limited regions, including the following: only men, pregnant women, children, the sickest, the most economically productive, individuals in the military, or even individuals of the dominant ethnic group [2]. It has also been proposed that a lottery would be the only fair approach to allocating ARVs [3]. Only a limited number of ARVs will be available, and only a fixed number of health-care facilities (HCFs) can be used for ARV distribution. Thus, the resource allocation decisions that need to be made are extremely complex.

Here, we use operations research to address this important resource allocation problem and to design ARV allocation strategies that are rational and equitable. The allocation decisions that we make here are based on ethical criteria, and not on epidemiological or preferential treatment priority criteria. Specifically, we determine the optimal allocation strategy that would ensure that each individual with HIV has an equal chance of receiving ARVs. We present a novel spatial mathematical model of treatment accessibility that we use in conjunction with an equity objective function to determine an optimal equitable allocation strategy (OEAS) for ARVs in a resource-constrained region. We quantify how changing the size of the catchment region surrounding each HCF, and the number of HCFs utilized for ARV distribution, alters the OEAS. Specifically, we use data from the detailed ARV rollout plan designed by the government of South Africa to determine an OEAS (based upon a variety of assumptions) for the province of KwaZulu–Natal. We also discuss how our proposed ARV allocation strategy differs from the currently proposed plan.

**Figure 1:**
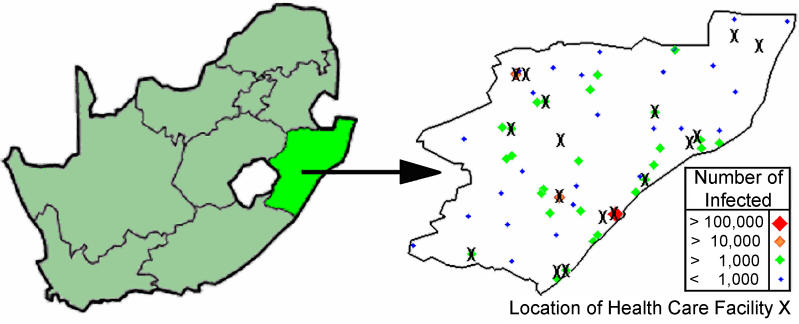
Map of South Africa Indicating the Location of the KwaZulu–Natal Province and Map of KwaZulu–Natal Black crosses indicate the location of the 17 HCFs that have been designated for ARV rollout by the South African government, and the spatial distribution of communities distinguished by the number of individuals infected with HIV (by both size and color). Durban (represented by the large red diamond) is the capital city of the province and has more individuals with HIV than any other community. Pietermaritzburg and Newcastle (represented by orange diamonds) have the next greatest numbers of individuals with HIV.

**Figure 2:**
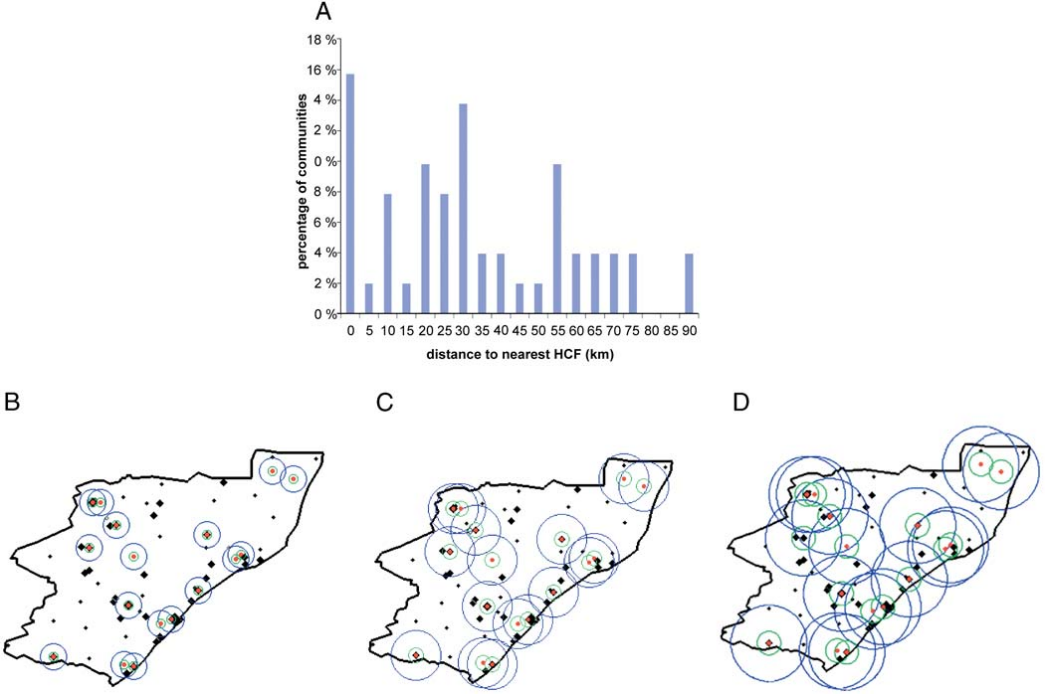
Accessibility of Communities to HCFs (A) A histogram indicating heterogeneity in the distance from communities in KwaZulu–Natal to the closest HCF. The treatment accessibility function used in our model is a Gaussian distribution, exp(−*kd* ^2^), indicating that accessibility is strongly related to distance *(d),* and *k* is a dispersal length scale parameter. (B) The catchment region is shown with an effective radius of 20 km for coverage from each HCF (*k =* 0.0151). (C) The catchment region is shown with an effective radius of 40 km for coverage from each HCF (*k* **=** 0.003786). (D) The catchment region is shown with an effective radius of 60 km for coverage from each HCF (*k* = 0.00168). In each case, the red dots indicate the location of the HCF, the green circles represent the locations where treatment accessibility has been reduced to 50% relative to someone located at the HCF, and the blue circles represent the locations where treatment accessibility has been reduced to 1% relative to someone located at the HCF. The locations of communities are presented as black diamonds. The large black diamonds denote large communities (with population greater than 10,000 people), and the small black diamonds denote small communities (with population less than 10,000 people). Substantially more area of the province is covered if HCFs have catchment regions of 60-km radius, relative to catchment regions of 40-km radius, and substantially less area of the province is covered if HCFs have a catchment region of only 20-km radius. However, the proportion of people with access does not differ greatly between the different catchment sizes because of the great spatial heterogeneity in the prevalence of people with HIV.

Our current analysis is applied to the South African province of KwaZulu–Natal, although our methodology could be applied to any resource-constrained setting. KwaZulu–Natal is the largest province in South Africa with a population of approximately 9.4 million and has more people infected with HIV than any other province (approximately 21% of all cases in South Africa [4]). We use data from 51 communities (cities, towns, and villages) in the province of KwaZulu–Natal; we exclude communities with a population of less than 500 people. Data are not available on the number of individuals with HIV in each specific community, and thus we use the estimated HIV prevalence in the region (approximately 13% in urban areas and 9% in rural areas [4]) to estimate the number of infected people in each community. See Figure 1 and Table 1 for the population sizes and spatial locations of each of the 51 communities used in our analysis. For our analysis the quantity of ARVs available for distribution to the HCFs is sufficient to treat 10% of the total number of infected people, which is a realistic level during the incremental scale-up of ARV therapy over the next few years. The government of South Africa has selected 17 HCFs to participate in the ARV rollout that began in April 2004. These 17 HCFs are distributed throughout the province (see Figure 1 and Table 2). Some communities are close to HCFs, whilst others are a great distance from any HCF, with a range of 0–90 km (Figure 2A). Hence, this spatial distribution of HCFs produces large heterogeneity in accessibility to treatment. Inequality in access to health care is a common characteristic of resource-constrained regions [5,6,7,8,9,10]. We explicitly consider heterogeneity in treatment accessibility in our analysis of ARV allocation strategies.

**Table 1:**
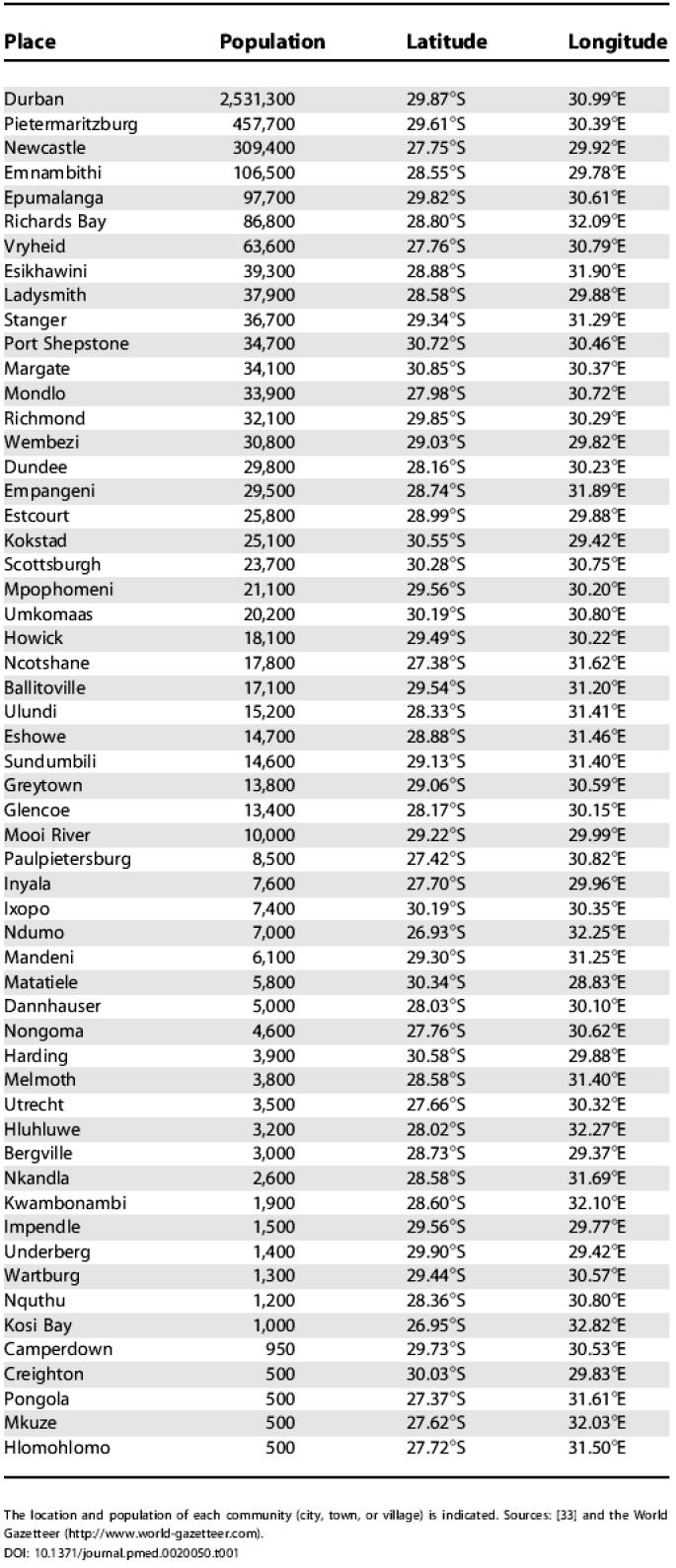
Communities in KwaZulu–Natal with Populations of at Least 500 People

The location and population of each community (city, town, or village) is indicated. Sources: [33] and the World Gazetteer (http://www.world-gazetteer.com)

**Table 2:**
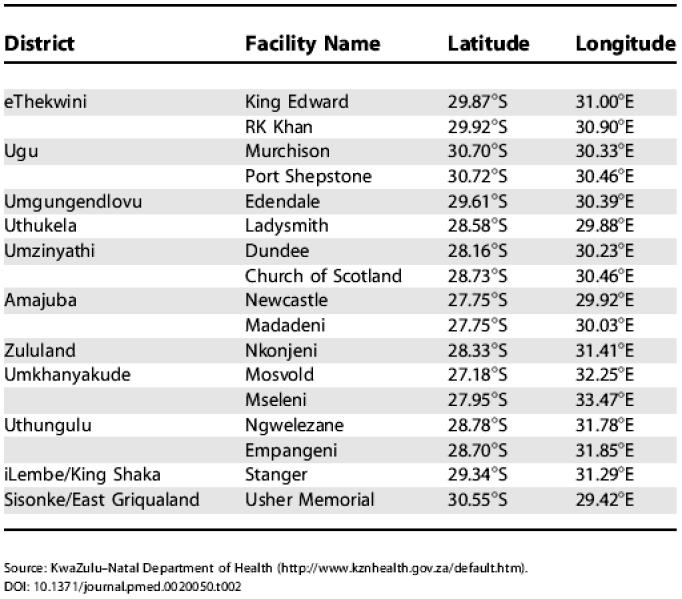
HCFs Proposed for Use in the Rollout of ARVs in KwaZulu–Natal

Source: KwaZulu–Natal Department of Health (http://www.kznhealth.gov.za/default.htm)

We have developed a novel spatial mathematical model of treatment accessibility that we use to determine an OEAS for ARVs in a resource-constrained region. To the best of our knowledge, this is the first analysis to address how to deal with the extremely difficult problem of allocating a scarce supply of ARVs in order to design a rational and equitable allocation strategy. We model the “spatial diffusion of treatment” to the locations of disease, rather than modeling the “spatial diffusion of disease,” which is the conventional approach [11,12,13,14,15,16]. Our spatial model includes HCFs and the HIV-infected communities surrounding these HCFs; we refer to the region around each HCF as the catchment region. Thus, the radius of the catchment region specifies the approximate maximum distance that we assume infected people would be willing (or able) to travel for treatment. Each HCF can serve many communities, and some communities can access multiple HCFs; our model sums the number of people with HIV in each HCF's catchment region who could potentially travel to the HCF to receive ARVs (we define this number as the “effective demand” on that specific HCF). Thus, the “effective demand” on each HCF is a direct function of the number of individuals with HIV in the catchment region, weighted by their distance from the HCF. By including a weighting function we explicitly model heterogeneity in accessibility to treatment based on distance from the HCF. Here, the distance from a HCF becomes the main determinant influencing whether or not an individual with HIV has access to treatment.

We developed an equity objective function to assess how the limited supply of ARVs should be allocated to each HCF to ensure that an equal proportion of infected people in each community receive treatment. To apply our theoretical framework to KwaZulu–Natal we model the specific location of the 17 HCFs and the 51 communities of 500 or more individuals (see Figure 1); for these conditions we determine an OEAS. We compared our OEAS with two other allocation strategies: (i) allocating ARVs only to Durban, the major urban area (i.e., concentrating ARVs where there is the best health-care infrastructure) and (ii) allocating ARVs equally to all 17 HCFs. We conduct our analysis assuming three different radii of catchment regions: 20 km, 40 km, and 60 km. We then extend this analysis and recalculate the OEAS assuming that more than 17 HCFs are available to distribute ARVs. This analysis is useful because there is a second potential pool of 27 ARV-implementation HCFs in the South African operational plan for ARV rollout [17]. We analyze this case, in which 27 HCFs are utilized in the ARV rollout, and we also analyze how optimal ARV allocation would change if all 54 hospitals in KwaZulu–Natal were operational for the rollout of ARVs.

## Methods

### Calculating Demand and Treatment Access

We assume that the number of people with HIV who will travel to a specific HCF is directly proportional to the number of individuals with HIV in that particular community, but that the probability of an individual traveling to receive ARVs (i.e., the treatment accessibility) decreases with distance from the HCF. We define *d_i,j_* as the distance from community *i* to HCF *j, f*(*d_i,j_*) as a weighting function that determines the treatment accessibility to a HCF based upon distance *d_i,j_,* and *I_i_* as the number of people with HIV in community *i*. The distance, *d_ij_,* between community *i* and HCF *j* is based on the longitude (lon) and latitude (lat) of each location and is determined by



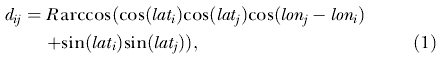



where *R* is the radius of the earth, taken to be 6,371 km, and the angles are in radian measure. We calculate the “effective demand” of community *i* on HCF *j* to be the number of people with HIV in community *i* that will travel to HCF *j* for ARV regimes, namely, *f*(*d_i,j_*)*I_i_*. Thus, demand on HCFs for ARVs is reduced by the treatment accessibility function. Our model is conceptually similar to the “gravity” models that have been used to predict retail travel [18], plan land use [19], and determine accessibility of primary care [20]. However, this is to our knowledge the first time this approach has been used to calculate ARV allocations. We use a Gaussian to model treatment accessibility, *f*(*d*) = exp(−*kd* ^2^), where *k* is a dispersal length scale parameter determining the radius of the catchment region. The size of the actual catchment regions is unknown, but based upon distances from communities to HCFs in KwaZulu–Natal (see Figure 2A) we assume that individuals are likely to travel a maximum distance of approximately 40 km to a HCF (*k* = 0.003786). We vary the catchment region by considering a 20-km radius (*k* = 0.0151) and a 60-km radius (*k* = 0.00168). The different catchment regions that we simulate (with radii of 20 km, 40 km, and 60 km) for each HCF are illustrated in Figure 2B–2D. The number of people with HIV throughout the province that have access to HCFs is approximately 86% of the total number of people with HIV for the case of a 20-km catchment region, 89% for a 40-km catchment region, and 93% for a 60-km catchment region.

### Modeling the Distribution of Treatment

To determine how many ARVs should be allocated to each HCF, we first calculate how a given supply of ARVs will be distributed from each HCF to the surrounding communities in the catchment region. We calculate the “effective demand” on HCF *j, D_j_,* to be



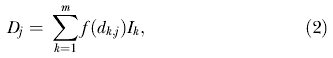



which sums the “effective demand” of all communities on HCF *j* (where there are *m* communities). Then, we model the distribution of ARVs from a HCF to each community within the catchment region as the proportion of the “effective demand” on HCF *j* that is contributed by the respective community. Accordingly, ARVs will be distributed from HCF, *j,* to each community as the ratio



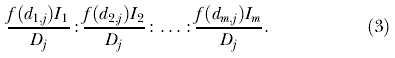



Therefore, the number of people treated in community *i* by the drug supply allocated to HCF *j* is



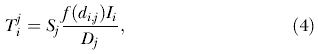



where *S_j_* is the number of regimes allocated to HCF *j*. Hence, the total number of people with HIV treated in community *i,T_i_,* summing over all *n* HCFs is



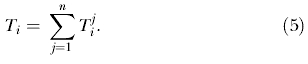



### The Equity Objective Function

We establish an equity objective function to determine the optimal equitable allocation of ARVs to each HCF so that all individuals with HIV have an equal chance of receiving treatment. To obtain the same fraction of treated individuals in each community, given that there are *A* ARV regimes for a total of 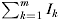 individuals with HIV, the resulting objective function to minimize (based on least squares) becomes



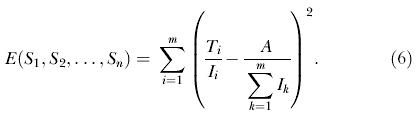



Our goal is to minimize *E,* by solving for the number of ARVs to be allocated to each HCF (*S*_1_, *S*_2_,…, *S*_n_), whilst enforcing the following three constraints: (i) ensure that the total number of ARVs available is equal to the sum of the supply allocated to all HCFs,



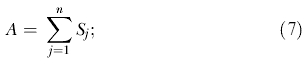



(ii) ensure that only a positive number of ARVs are allocated to each HCF (*S_j_* ≥ 0, *j* = 1…*n*); and (iii) ensure that the number of people treated in each community is not greater than the number of people with HIV in the community (*T_i_* ≤ *I_i_, i* = 1…*m*). We note that if a different objective is required, then all of our preceding analysis still holds and only the functional form of the objective function needs to be altered. To solve the problem, and determine the OEAS, we used successive linear programming operations research techniques [21].

## Results

**Figure 3:**
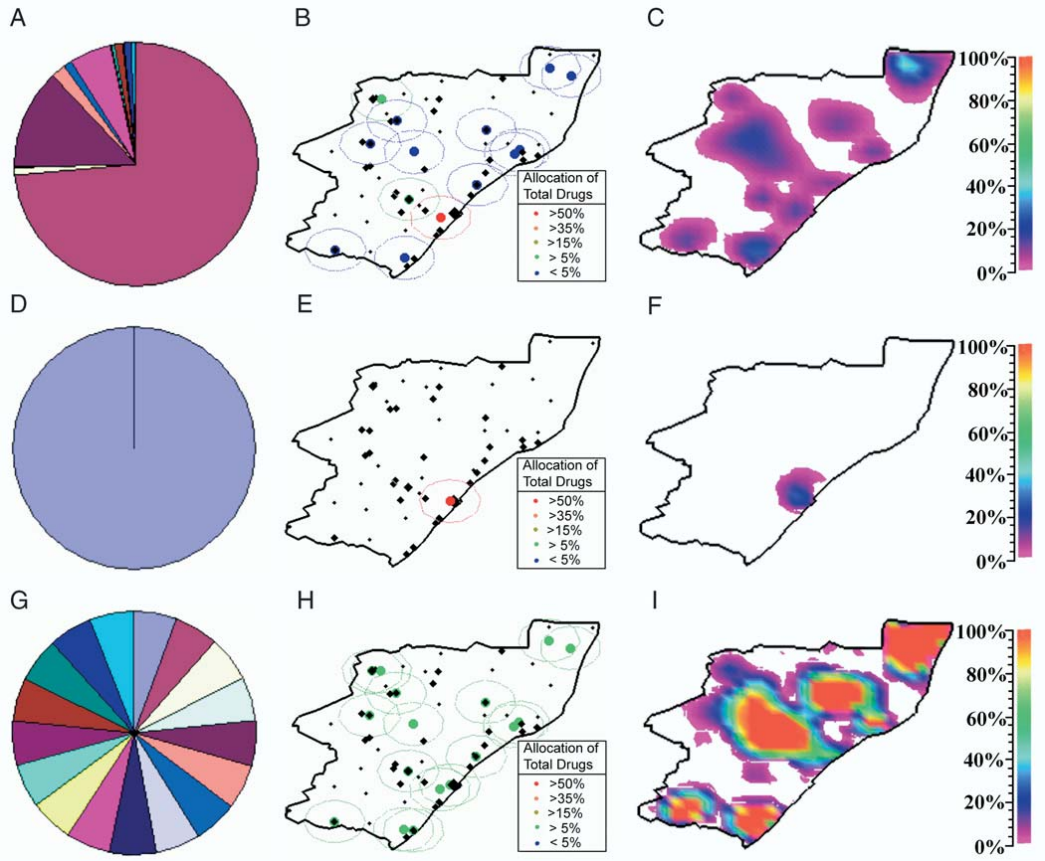
Pie Charts of the Three Strategies for Allocating ARVs to HCFs The three strategies considered are as follows: allocation of ARVs according to the results of minimizing our objective function (first row) allocation of ARVs only to one HCF in Durban (second row), allocation of ARVs equally to each of the 17 HCFs (third row). The proportion of ARVs allocated by these strategies to the 17 different HCFs is indicated in (A), (D), and (G), with each HCF represented by a different color. The spatial allocation of ARVs is shown in (B), (E), and (H), respectively. The respective percentage of infected people that are treated throughout the KwaZulu–Natal province is simulated in (C), (F), and (I). Here, the *x–y* plane represents spatial location, and the shaded color at a location refers to the proportion of individuals with HIV that are treated at the specified location. The plots were obtained by generating an interpolating surface where the *z*-ordinate, colored by magnitude, represents the proportion of treated individuals, and then orientating the view of the surface normal to the *x–y* plane. We performed surface data interpolation using the method of translates [32].

The OEAS of ARVs in KwaZulu–Natal that we determined is complex (see Figure 3A and 3B). According to our OEAS, the majority of ARVs should be allocated to HCFs in Durban, and the remaining ARVs should be allocated to the other HCFs throughout the province (with two non-Durban HCFs receiving 5%–15% of the total ARVs and the remaining non-Durban HCFs each receiving less than 5% of the total ARVs available). We note that our OEAS does not produce perfect equality; however, our optimal strategy significantly improves equality in obtaining treatment over the two other allocation strategies that we analyzed for comparison: (i) ARVs allocated only to one HCF (in the largest city, Durban) (see Figure 3D and 3E), and (ii) equal quantities of ARVs allocated to each HCF throughout the province (see Figure 3G and 3H). For comparison of allocation strategies (in Figure 3) we used an effective catchment radius of 40 km (*k* = 0.003786). The proportion of infected individuals that are treated at each location is displayed graphically in Figure 3 for our OEAS (Figure 3C) and the two comparison allocation strategies (Figure 3F and 3I). The best achievable outcome, given the limited treatment resources available, is that 10% of people with HIV are treated in each community throughout the province, yielding the map shown in Figure 3C, 3F, and 3I, but with dark blue/magenta over the entire province. Whilst our OEAS does not fully achieve this, it is considerably better than both of the comparison ARV allocation strategies. Furthermore, the equity objective function evaluates to *E* = 0.27 for our OEAS, compared with (i) *E* = 0.50 and (ii) *E* = 133.88 for the comparison allocation strategies. There is large diversity in the fraction of individuals with HIV treated per community when equal quantities of ARVs are given to each HCF, evidenced by an inter-quartile range of 0.025%–41.746% compared with inter-quartile ranges of 0%–0% and 0.011%–9.982% for the first comparison strategy and our OEAS, respectively. Therefore, equal access is not obtained if equal quantities of ARVs are allocated to each HCF. Obviously, allocating to only one HCF (the first comparison strategy) could also be considered unequal because although the inter-quartile range is minimal, effectively only one community (Durban) receives ARVs. Our OEAS, while not perfect, achieves the best equality possible given the accessibility constraints and limited ARV supply.

**Figure 4:**
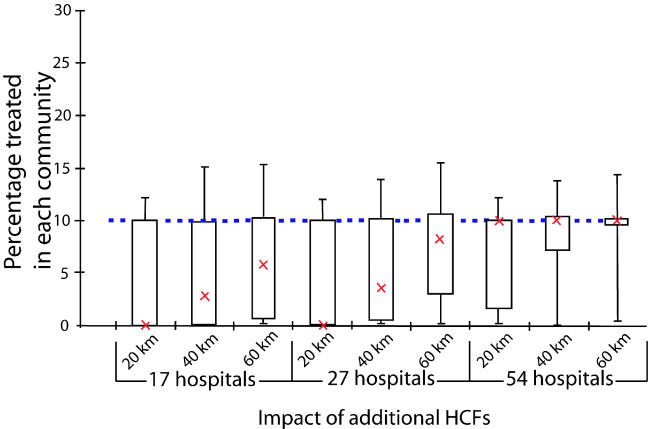
Percentage of People with HIV That Obtain Treatment per Community for Various Approaches Box plots of the percentage of infected people that obtain treatment per community for the three different sets of HCFs simulated in our analysis for ARV rollout, namely, using the 17 HCFs likely to be used, the 27 HCFs suggested by the South African government as potential implementation points, and all of the 54 hospitals in the KwaZulu–Natal province. These cases are represented for each of the three catchment region sizes we considered (with radii of 20 km, 40 km, or 60 km) and referenced against the ideal fraction treated (dotted blue line) under perfect conditions of egalitarian distribution, given the limited ARV supply. The red crosses indicate the median percentage of people with HIV that obtain treatment per community.

**Figure 5:**
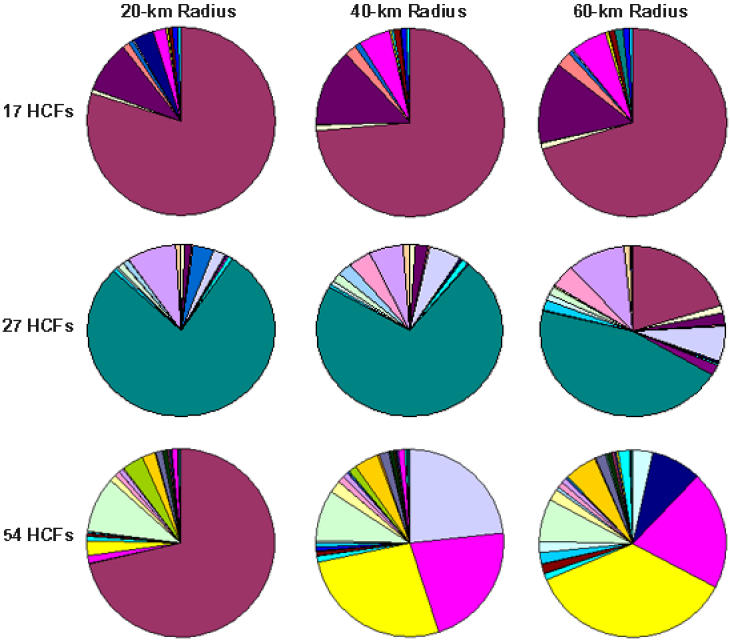
Actual Allocation of ARVs to HCFs These pie charts show ARV allocation to HCFs according to our model and optimization for the cases of 17 , 27 , and 54. The allocation is shown for each of the catchment region sizes considered: 20-km radius, 40-km radius, and 60-km radius.

The catchment region for HCFs is a factor of large uncertainty. We considered three catchment region sizes: radii of 20 km, 40 km, and 60 km. We also simulated two additional cases with increased numbers and locations of HCFs (27 HCFs as suggested in South Africa's official ARV rollout operational plan [17]; and all 54 hospitals in KwaZulu–Natal). In Figure 4 we present box plots of the percentage of infected people that obtain treatment per community for the three sets of HCFs and the three catchment region sizes we simulate. For each specified condition we calculate the OEAS. It is apparent that equality in access to ARVs is improved substantially if the radius of each catchment region is increased and/or the number of HCFs is increased (Figure 4). Our results show that the number of HCFs utilized is of greater importance than the size of the catchment region. If 54 HCFs are used, then even a (small) catchment radius of 20 km results in the ideal median proportion of 10% of people with HIV in each community receiving ARVs. In the case of 27 HCFs, 88% of all people with HIV have access to HCFs for a 20-km catchment region, 91% for a 40-km catchment region, and 96% for a 60-km catchment region. In the case of 54 HCFs, 90% of all people with HIV in the province have access to HCFs for a 20-km catchment region, 94% for a 40-km catchment region, and 99% for a 60-km catchment region. Therefore, increasing the number of HCFs available for an ARV rollout is effective in significantly increasing equality in treatment accessibility as shown in Figure 4. Furthermore, if catchment regions actually have a radius of 60 km, or can be increased to this size through improvements in transportation, this would enable access to HCFs for almost all people in the province, as shown in Figure 4. The actual HCF allocations determined by our model and optimization for the cases of 17, 27, and 54 HCFs (and for all catchment sizes we consider) are presented as pie charts in Figure 5. It is clear from our analysis that the equality criterion, such that each individual with HIV in KwaZulu–Natal has an equal chance of receiving ARVs, can best be satisfied by utilizing all 54 HCFs for ARV distribution and ensuring that each HCF serves a catchment region of 40 to 60 km.

## Discussion

We have established an elegant and simple theoretical framework for determining an equitable and rational allocation of ARVs to HCFs in resource-constrained countries. To the best of our knowledge, this is the first analysis to address this very difficult problem. We determined that increasing the size of the catchment region of each HCF can improve access to HCFs considerably for rural populations. We suggest that studies be performed to collect data on the distance that individuals with HIV are willing and able to travel for treatment. This will facilitate discussions of this important issue, which must be considered in the making of policy decisions. A database consisting of such information has been proposed for South Africa [22]. In an effort to provide equal access to communities with relatively little access to ARV therapy, the concept of a mobile clinic that would travel between communities to take health-care workers and resources to the location of the demand is a new initiative in Nigeria (S. Agwale, personal communication) that could also be considered in other regions.

We calculated the optimal allocation of ARVs to available HCFs so that all infected individuals will have as close as possible to an equal chance of obtaining treatment. We have shown that increasing the number of HCFs involved in ARV distribution can improve equality of access to ARVs substantially. The current plan in KwaZulu–Natal is to use only 17 HCFs. However, our results clearly show that in order to achieve an optimal equitable allocation strategy, all existing infrastructure (i.e., all 54 HCFs) should be used. The strategy that we are advising may be fairly easy to accomplish at the policy level because the health-care infrastructure (specifically these HCFs) already exists, although consideration must be made for issues such as the training and transportation that is necessary, which may be costly. In contrast, increasing the size of catchment regions may be very difficult. Obviously, increasing both the number of HCFs and the size of the catchment region each services would substantially increase equality of access to health care in KwaZulu–Natal.

Future modeling studies could extend our work by not making the simplifying assumption that all patients have similar ease of travel over the same distance and by including weighting functions on distance impedance for different communities (based on the quality of the road infrastructure, for example, and the availability of transportation) (D. P. Wilson, J. O. Kahn, S. M. Blower, unpublished data). Here, we have shown how to calculate optimal ARV allocation strategies based upon the principle of equity. Future research is necessary to compare ARV allocation strategies based upon the principle of efficiency (i.e., allocating ARVs to maximize epidemic reduction) in order to determine whether utilizing different principles for optimization would result in similar (or different) allocation strategies.

The World Health Organization and the Joint United Nations Programme on HIV/AIDS have identified three core principles that should underlie the effort to fairly distribute ARVs, namely: urgency, equity, and sustainability [23]. They state that policy decisions for the fair distribution of ARVs should be based upon the following ethical principles: (i) the principle that like cases should be treated alike, (ii) the utilitarian principles of maximizing overall societal benefits, (iii) the egalitarian principles of equity (distributing resources, such as health care, equally among different groups), and (iv) the Maximin principle (which prioritizes individuals that are the least advantaged) [24]. Here, we investigated the level of decision-making associated with allocating ARVs to HCFs, and we have applied the egalitarian principle of equity with respect to access to health care. We suggest that allocating ARVs to HCFs to achieve equality in accessibility could be carried out, and then individual-level ethical considerations could be thought out at the next level of deliberation. Future research is necessary to identify alternative (and more detailed) ethical ARV allocation strategies.

Although we have focused on one equitable strategy, there are many other ARV allocation strategies that are ethical. Uneven access to HIV treatment has the very real potential to fracture social and political structures and could lead to intrastate and/or interstate conflict [2]. Government decisions on ARV allocation have potentially socially destabilizing ramifications because essentially the decisions determine who lives and who dies. Resource allocation decisions will have to be made at a number of levels: it must be decided what proportion of the available ARVs should be allocated to each province; then it must be decided how many ARVs should be allocated to each HCF within each region; and finally, particular groups of individuals may be chosen to have treatment priority.

Treatment priority decisions for individuals could be based on many different criteria, including disease progression (CD4 cell counts and viral load), socioeconomic status, ethnicity, and who is thought to have the greatest risk of transmitting infections (for example, pregnant women with HIV or female sex workers). Although it could be argued that behavioral core groups should be targeted to receive ARVs because this may have the greatest epidemiological impact, such an allocation strategy would be neither feasible nor practical to implement. For example, sex workers are an obvious behavioral core group, but many women would likely claim to be sex workers if they knew that ARVs were only available to sex workers. Additionally, the ethics of targeting such groups in favor of other societal groups must be questioned. It could also be argued that, to maximize the preventative effect of ARV therapy, ARVs should be concentrated in virological core groups (i.e., people with the highest viral load) [25,26]; this novel approach of targeting the virological core group has recently been proposed for controlling HSV-2 epidemics [27]. Identifying individuals in the virological core group would be far easier than identifying individuals in the behavioral core group. These individuals are likely to be the sickest and those with evidence of disease-related symptoms. Treatment allocation strategies could also be designed based on reducing the future epidemic impact and disregarding treatment equality amongst currently infected people. Such strategies place different social value on currently infected people in comparison with future infected people; such strategies therefore may not be ethical even though they may be epidemiologically sound (also, it is important to note that any epidemic predictions have large uncertainty ranges [28,29]).

Our model has been applied to the South African province of KwaZulu–Natal, but it can be applied by government health officials in any resource-constrained country. In many of the countries worst affected by the HIV pandemic, scarcity of resources will mean that not everyone that could potentially benefit from ARVs will be able to access them. Many of the decisions that must be made to develop an effective response to the HIV/AIDS epidemic are inevitably underpinned by ethical considerations. Leadership in most resource-constrained regions cannot avoid these decisions. Whilst there has been considerable attention given to South Africa, many other countries worldwide either have plans in place (e.g., Brazil, Thailand, and Botswana) or are in the process of developing national programs for ARV distribution through the public health system (e.g., Mozambique, Malawi, and Kenya) [1]. Legitimate authorities in each nation must come to their own consensus on the priorities and objectives of an ARV rollout, which is not a trivial matter [1,30]. Our objective function and model can be used to calculate allocation strategies that provide equity in access (compensating for geographical isolation), but if authorities in a given nation prioritize a different goal for ARV rollout, then an objective function to optimize can be formulated to reflect the specific national policy goal. Our model can be used by policy makers to determine an optimal scientifically based allocation strategy, based upon the specific objective function. As the ARV rollout commences in KwaZulu–Natal, difficult decisions will have to be made as to how to allocate scarce resources. We have shown that it is possible to obtain a mathematical solution to an equity problem. We suggest that our novel approach could be used to determine optimal equitable allocation strategies for many other resource-constrained countries that are just beginning to receive ARVs [31].

**Patient Summary**

**Background**

Antiretroviral drugs can change the lives of patients with HIV/AIDS. Their high price, however, means that many poor countries do not have enough of these drugs to treat all the people who need them. The decision of who will get treatment is very difficult, and different ways to come up with ethical solutions to the problem have been proposed.

**Why Was This Study Done?**

One of the approaches is to try to make sure that every infected person has the same chance to get antiretroviral drugs. David Wilson and Sally Blower, the authors of this study, wanted to find a scientific strategy to achieve this goal of equal access.

**What Did the Researchers Do?**

They used mathematical models to calculate how to distribute available drugs among hospitals and doctor's offices so that each patient in a particular area had an equal chance to get treated.

**What Did They Find?**

When they used their approach on a real example, the South African province of KwaZulu–Natal, they found that making some changes to the current plans for drug distribution would lead to more equal access among all of the individuals with HIV in the province. Instead of only 17 out of the 54 health care facilities in KwaZulu–Natal distributing the drugs (which is the current plan of the South African government), Wilson and Blower calculate that it would be fairer if all 54 facilities distributed the medicines.

**What Does This Mean?**

Mathematical models like the one used here are always based on assumptions and simplifications. As a consequence, they are never perfect matches for a real-life situation, but they can help to guide complicated decisions. This article suggests that the approach Wilson and Blower developed could help to determine strategies for equitable allocation of limited HIV treatment resources.

**What Next?**

The authors hope that the tools they developed will be used by policy makers in resource-poor countries to guide their strategies. They are keen to work with these policy makers to adapt and optimize the method to local settings and priorities.

**More Information Online**

Report by the World Health Organization and the Joint United Nations Programme on HIV/AIDS on ethics and equitable access to HIV/AIDS treatment: http://www.who.int/hiv/pub/advocacy/en/ethicsmeetingreport_e.pdf

Ruth Macklin's report on ethics and equity in access to HIV treatment: http://www.who.int/ethics/en/background-macklin.pdf

The Pro-Poor Health Policy Team's report on priority in HIV/AIDS treatment: http://www.who.int/ethics/en/background-pro-poor3.pdf

News article from the World Health Organization Bulletin on the South African HIV/AIDS treatment program: http://www.who.int/bulletin/volumes/82/1/en/news.pdf

## Abbreviations

ARV: antiretroviral
HCF: health-care facility
OEAS: optimal equitable allocation strategy
